# Donkin psychosis

**DOI:** 10.1007/s00737-016-0677-6

**Published:** 2016-10-08

**Authors:** Ian Brockington

**Affiliations:** University of Birmingham, Edgbaston, Birmingham,, B15 2TT UK

**Keywords:** Donkin psychosis, Eclamptic psychosis, Seizures, Lucid intervals

## Abstract

Donkin psychoses are eclamptic psychoses without seizures. As symptomatic psychoses resulting from cerebral endothelial damage, they may explain the lucid intervals that sometimes occur between eclampsia and the eruption of psychosis. They have the same features as eclamptic psychoses, with onset during pregnancy or the early puerperium, especially in first-time mothers, a short duration and full recovery in most. The clinical picture is usually delirium, but mania is also seen, and some patients have retrograde amnesia or other cognitive defects. Donkin psychosis should be considered in the differential diagnosis of childbearing psychoses, and collaborative research is needed to clarify their differences.

## Introduction

Eclamptic psychosis was first described in the seventeenth and eighteenth century in Spain (Mercatus 1614) and Germany (Boenneken 1744). It was rife in the 1850s when Simpson, following the lead of Bright, who discovered protein in the urine of patients with nephritis, started measuring albuminuria and found it in cases of puerperal psychosis (Simpson 1857). Its role was exaggerated, just as in the golden age of bacteriology, the role of infection was later exaggerated (Hansen 1888). One of Simpson’s students was Arthur Donkin, who became a Lecturer in Medical Jurisprudence at Newcastle. In 1863, he described this case:

A 23-year old gave birth to twins. Her face was puffy and oedematous, and her urine contained albumin. On day 1 she became agitated and complained of uneasiness and lightness in the head, photophobia, drumming in the ears, noise intolerance and insomnia. On day 3 she became excited, violent and maniacal, and required restraint. She insisted there was another baby to come, and it would be necessary to cut open her womb. With brief remissions, delirium and excitement continued until day 12. She then recovered.

This is eclamptic psychosis *sine* seizures. Although one can, with hindsight, identify earlier cases (Smith 1851, Jenkins 1857), Donkin recognized its eclamptic origin. This case aroused great interest on the continent (Olshausen 1891, Hoppe 1893, Évrot 1894), even though it has never previously been cited by a British author.

## Cases in the literature

### Typical Donkin cases

Forty years later, a second example was published (Lauly 1904):

A 31-year old developed oedema and albuminuria during her first pregnancy. On October 7th, she gave birth. On the 14th she developed headache and confusion, and was continuously muttering, in a dream-like state with visual hallucinations. Her temperature was 39.5° and albuminuria, which had disappeared after the birth, reappeared. She said goodbye to her baby, because she was going to heaven. Her confusion became more intense and hallucinatory. Admitted to hospital, she remained irritable & confused until November 11th (28 days), with partial amnesia for the illness.

These cases are a manifestation of the wider spectrum of eclamptic illness, which, even in fatal cases, need not include seizures. The central nervous manifestations are kaleidoscopic, including jerking in the face and limbs, blindness, apathy, stupor, coma, or twilight and dream-like states (Kehrer 1924). The idea of psychosis as ‘eclamptic equivalents’ took root (Engelhard 1912, Rochaix 1913, Sioli 1924).

The literature contains 15 other cases similar to Donkin’s—three prepartum (McGeorge 1934, Visscher 1949, Bourson 1958), three starting during labour or immediately afterwards (Jolly 1911, Bernard 1922, Cimellaro 1962), one after a 5-month termination of pregnancy (Engelhard 1912) and eight after the birth (Engelhard 1912, Bernard 1922, Cruchet & Rivière 1923, Wu 1933, Atkin 1938, Moschel 1965, Gödtel 1965, Marzotko 1967). It is not surprising that all these cases were described in the twentieth century, because after the clinical measurement of the arterial pressure became possible (in 1896), the diagnosis of pre-eclamptic toxaemia became easier.

To understand the significance of Donkin psychoses, two other features of eclamptic psychosis are important—onset of psychosis before seizures and the gap between seizures and onset of psychosis.

### Onset of eclamptic psychosis before seizures

In the literature, there are seven cases of eclamptic psychosis (five prepartum and two postpartum) in whom psychotic symptoms preceded the first convulsion (Scanzoni 1852, Jolly 1911, Engelhard 1912, Long 1936, Ciulla 1940). These two American cases are the best examples:

A Maryland woman complained of headache at term. Immediately after the birth, when the placenta was lying in the vagina, she became ‘a complete maniac’. Potassium bromide and five hours anaesthesia with chloroform had no effect on her raving and mental excitement. It required two attendants to keep her in bed. About 50 hours after the birth and onset of mania, she had a convulsion, after which she lay perfectly quiet. She had further fits at intervals of about an hour, until 12 hours after the first seizure, she had her last and most violent fit. She then improved rapidly (Maynard 1872).

A 26-year old vomited until two weeks before the delivery of her 1^st^ child. She developed swelling of the hands and legs and, suddenly, enormous swelling of the vulva. On March 16th labour was induced, and a live baby born. On day 1 she complained of headache and dim vision, and was found to have albuminuria. She was wildly delirious and hard to restrain. Her blood pressure was 178/120. On day 2 she had six convulsions. She was treated with potassium bromide 60 grains/6 hourly. She was unconscious until day 5. On day 6 she thought her mother had died and could hear voices in the room, including her sister calling her. On days 7-9 she was restless and screaming, or muttering incoherently; she then rapidly recovered, with amnesia from the first convulsion (Cornell 1919).

It is unlikely that unobserved seizures occurred before the eruption of mania, because Cornell’s patient was already in hospital.

This temporal dissociation of psychosis and seizures indicates that there are two separate cerebral complications of gestosis—seizures (with post-epileptic confusion) and a symptomatic psychosis. Donkin’s patient had one, not the other.

### Lucid intervals between seizures and psychosis

In many cases, eclamptic psychosis begins as soon as the patient emerges from post-convulsion coma or stupor. These are perhaps equivalent to post-epileptic confusional states. But in some mothers, psychoses start after a ‘lucid interval’, as in this first description (Gooch 1829):

A *primipara* was seized with convulsions during labour. The child was delivered by perforating the head. The convulsions ceased and for several days she seemed to be doing well. But a few days after delivery, she became maniacal and died three days later, eight days after the birth.

Several authorities have emphasized this lucid interval (Olshausen 1891, Hoppe 1893, Quensel 1907). Three German authors provided data on its frequency: in a series of 32 cases, ten had an immediate onset of psychosis, 18 after an interval of 1–6 days, three a week or more afterwards, and one 20 days after the last seizure (Kutzinski 1909). In a series of 60, most began 2–3 days after the onset of eclampsia, and ten more than 6 days later, up to a limit of 16 days (Herrmann 1929). The last author recorded the interval from the first fit to the end of coma in 26 cases: it ranged from a few hours to 12 days (Kleinknecht 1914). The following Italian and Argentinian cases had the longest lucid intervals (Franchini 1955, Lértora 1955):

A 26-year old was delivered by forceps in the 8th month of her 1st pregnancy, with eclamptic fits. Fifteen days later she presented with aphasia, confusion, retardation and ideas of ruin. She had heavy albuminuria. She recovered in eight days.

A 40-year old, in the 6th month of her 2nd pregnancy, suddenly had a seizure. Her systolic blood pressure was 220, and she had albuminuria. Four days later (March 13th) she gave birth to a dead child. On 29th, she suddenly became disorientated and confused, then mute. After a pneumo-encephalogram, she died.

These intervals seem too long for post-epileptic confusion. An alternative explanation is that they are Donkin psychoses, occurring in mothers who also suffered seizures. With this adjustment, I have identified 46 Donkin cases. Their distribution by generation is shown in Table 1.

**Table 1:** Distribution of eclamptic and Donkin cases by generation

Generation	Eclamptic psychosis	Donkin psychosis	Ratio
Before 1825	9	None	230/35
1826–1850	20	2	
1851–1875	29	6	
1876–1900	56	1	
1901–1925	90	18	
1926–1950	29	8	
1951–1975	7	8	10/11
After 1975	3	3	
Total	243	46	

The increased ratio after 1950 is highly significant statistically (*p* < .0001). Donkin cases were much less common in the early literature. This suggests that the diagnosis was being missed. If the ratio after 1950 is correct, at least 100 other cases are concealed in the early literature, In an era when there were many reports of psychoses that cleared up within a few days (much too soon for bipolar/cycloid disorders).

### Clinical features

The clinical features shared by eclamptic and Donkin psychoses include the following:

- They occur in about 5 % of eclamptic cases or more when seizures begin postpartum.
- There is a preponderance in first time mothers (eclamptic 59 %, Donkin 53 %).
- Usually, the psychosis develops after the birth, but 22 mothers had a prepartum onset (17 in the 3rd trimester and 5 in the 2nd trimester).
- In postpartum cases, the onset of psychosis is early, with a median of day 3 or (with a lucid interval) day 7.
- Brevity is a mark of these psychoses: the modal duration is 3–4 days. The median for eclamptic psychosis is 8 days, and for Donkin psychoses 14 days.
- The clinical picture is usually that of delirium, but as in infective psychoses, (Brockington 2014), manic disorders are also seen (Sélade 1847/8, Seydel 1868, Senlecq 1889, Sioli 1924, Bourson 1958, Gödtel 1965)
- Cognitive defects such as retrograde amnesia, dysphasia, and hemiplegia.

Since onset and duration are important elements, Table 2 shows data in patients with sufficient information:

**Table 2:** Onset and duration of eclamptic and Donkin psychoses

Diagnosis	Onset				Duration^a^		
	Pregnancy	Labour	Days 1–10	>10 days	<14 days	14-28 days	>1 month
Eclamptic psychosis	21	9	133	3	107	25	34
Donkin psychosis	6	2	26	8	17	2	14

^a^This duration is for survivors; 23 mothers with eclamptic psychosis and 7 with Donkin psychoses died

Onset after day 10 is unusual, but there are seven cases that started on day 11 (Kutzinski 1909, Clauser 1922), day 13 (Kitabayashi *et al*. 2007), day 15 (Franchini 1955), day 16 (Sachs 1910), and day 17 (Gueye 1976); this is the extreme example of late onset, on day 41, but with transitory agitation and clouding on day 4:

A 27-year old, in the 7^th^ month of her 1^st^ pregnancy, developed headache, dyspneia and albuminuria. After the birth, the placenta was noted to have infarcts. On day 4 she was agitated and clouded and believed she would die. On day 41 she developed *délire*, with confusion, retardation, hallucinations of coffins, paraesthesiae & loss of memory. She recovered after five months (Rochaix 1913).

These cases give qualified support to the idea that these psychoses can present later than the second week. As for duration, there is no doubt that a minority last for a relatively long time—48 (21 %) for over a month and 18 (10 %) for more than 3 months to a maximum of 9 months.

### Personal experience

Among the 321 cases of childbearing psychosis in my series, 13 mothers (4 %) *may* have suffered from Donkin psychosis, although none was typical. This is an example:

A 30-year old was pregnant for the first time. She developed a high blood pressure (140/90) and was admitted to hospital five weeks before the expected date of delivery because the baby was small; a Doppler test showed a grossly abnormal placenta. She said she had swelling of the face (not recorded in the records). A few days later, at 37 weeks gestation, she was delivered by Caesarean section of an infant weighing 4 lb 8 oz. Three weeks after the birth she became sleepless and irrationally anxious; for example, she was afraid of drowning in the shower. She woke up, convinced the baby was dead, screamed and tried to escape. She believed she was dying, was living in another world and had various and multiple illnesses; her parents and sister were dead. She accused a friend of having an affair with her father, and thought the house was haunted and on fire. She attacked her husband and believed she had killed him. On admission to hospital, she thought the ambulance men were police and she was being imprisoned. She appeared anxious, perplexed and confused, but was fully orientated. She had various auditory hallucinations. She could smell the hospital ward on fire and hear the crackling. She soon recovered and was discharged three weeks after the onset of the psychosis.

The onset was at the limit observed in the literature, but the duration was short for a bipolar/cycloid episode and there was no evidence of bipolar disorder in her family or the next twenty years.

Until the differential diagnosis has been clarified, it would be unwise to assume that any of these patients with severe PET suffered from puerperal bipolar disorder.

## Discussion

Eclampsia (gestosis) is an endothelial disease, due to placental abnormalities. The prodromal state—pre-eclamptic toxaemia—has been brought under control in nations with high obstetric standards, but still occurs: in a recent survey of over 275,000 women in 24 nations, it had a prevalence of 4 % (Bilano *et al*. 2014). At the beginning of the twentieth century, eclampsia complicated 2–3 % of pregnancies (Kleinknecht 1914). Better diagnosis and antenatal care has reduced this to about 1/500–1/1,000 pregnancies (Cunningham & Twickler 2000). But the frequency is still high in the developing world, for example India (Bathla *et al*. 2002) and Nigeria (Obed *et al*. 1997). Indeed, in Kolkata, the astonishing rate of 7.4 % was reported in 70,000 *primigravidae* (Pal *et al*. 2011). In Mexico, Brazil, South Africa and Gabon, it is the commonest cause of maternal death (Veloz-Martinez *et al*. 2010, Vega *et al*. 2007, Moodley 2010, Mayi-Tsonga *et al*. 2008), and in Bangladesh the second highest after haemorrhage (Halim *et al*. 2014). Most of these reports do not mention psychosis, but small series were reported from Delhi (Bathla *et al*. 2002) and Maiduguri (Obed *et al*. 1997). In these nations, eclamptic and Donkin psychoses will be common.

Gestosis is still a disease of unknown cause. There are widespread renal and hepatic lesions. In the brain, they are found in 60 %, ranging from microscopic haemorrhages and petechiae to large cerebral bleeds together with white matter oedema (Hinchey *et al*. 1996), especially in the posterior parietal, temporal and occipital regions (‘reversible posterior leukoencephalopathy syndrome’). The vasomotor disturbance that underlies seizures and coma usually leaves no residue. Thus, ‘toxaemia’ or gestosis appears to be a widespread arterial disease, with ischaemic lesions due to a transient but intense vasoconstriction.

Eclampsia is uniquely related to pregnancy and cured only by delivery of the placenta. Its increased frequency in hydatidiform mole shows that the foetus is not responsible. Page (1939) suggested that the placenta—‘a ruthless parasitic organ’—was the agent, secreting a pressor in response to ischaemia. Its role was demonstrated by a case of abdominal pregnancy, where the infant was surgically removed, leaving part of the placenta, and the mother experienced a worsening of pre-eclampsia for 2 weeks (Shembry & Nobel 1995). Recent papers (Roberts 1998, Vatten & Skjaerven 2004) have assembled the evidence of placental dysfunction: various placental anomalies predispose—multiple pregnancy, excessive placental size or abnormal implantation. Roberts has suggested that the placental pressor targets the endothelium, which is not, like cellophane, a barrier between blood and the collagen of the vessel wall, but a complex organ with numerous functions; the consequences include arteriolar spasm and activation of the coagulation cascade, causing microthrombosis. He has also (Roberts 2014) suggested that the primary abnormality is a failure to remodel the spiral arteries in early pregnancy. In the last 20 years, biochemists have demonstrated, in PET, changes in endothelin, thromboxane and thrombomodulin, and an increase in anti-angiogenic proteins secreted by the placenta; these may be the cause of the widespread damage to the maternal endothelium (Granger *et al*. 2001, Roberts 1998, Nader *et al*. 2004, Steinberg *et al*. 2009).

If as many as 4 % of childbearing psychoses, seen in Britain in this era, are Donkin psychoses, this could introduce an unwelcome heterogeneity into samples used for neuroscientific research (Brockington. in press). It is important to recognize the possibility and to tackle the diagnostic uncertainty. The difficulty with Donkin psychosis, as with other nosological concepts in psychiatry (including mania), is that the intension (essence) is clear, but the extension (boundary) is not clear at all. This problem may not be soluble at the clinical level—it may require EEGs and laboratory tests. In consultation with experts, it may be possible to use laboratory findings to indicate the severity of the gestosis, and help define the mental disorder Donkin discovered.
